# Effect of Octreotide Long-Acting Release on Tregs and MDSC Cells in Neuroendocrine Tumour Patients: A Pivotal Prospective Study

**DOI:** 10.3390/cancers12092422

**Published:** 2020-08-26

**Authors:** Claudia von Arx, Giuseppina Rea, Maria Napolitano, Alessandro Ottaiano, Fabiana Tatangelo, Francesco Izzo, Antonella Petrillo, Ottavia Clemente, Antonella Di Sarno, Gerardo Botti, Stefania Scala, Salvatore Tafuto

**Affiliations:** 1 Department of Clinical and Experimental Thoracic oncology Istituto Nazionale Tumori, IRCCS Fondazione G.Pascale, 80131 Naples, Italy; claudia.vonarx@gmail.com; 2 UOC Bersagli Molecolari del Microambiente, Istituto Nazionale Tumori, IRCCS Fondazione G. Pascale, 80131 Naples, Italy; pina.rea@hotmail.it (G.R.); m.napolitano@istitutotumori.na.it (M.N.); s.scala@istitutotumori.na.it (S.S.); 3 Department of Abdominal Oncology, Istituto Nazionale Tumori, IRCCS Fondazione G. Pascale, 80131 Naples, Italy; a.ottaiano@istitutotumori.na.it (A.O.); f.izzo@istitutotumori.na.it (F.I.); ottaviaclem@gmail.com (O.C.); 4 Department of Pathology, Istituto Nazionale Tumori, IRCCS-Fondazione G. Pascale, 80131 Naples, Italy; f.tatangelo@istitutotumori.na.it; 5 Department of Diagnostic Imaging, Radiant and Metabolic Therapy, Istituto Nazionale Tumori, IRCCS Fondazione G. Pascale, 80131 Naples, Italy; a.petrillo@istitutotumori.na.it; 6 Department of Internal Medicine, AORN dei Colli, Ospedale “A. Monaldi”, 80131 Naples, Italy; antonella.disarno@gmail.com; 7 Scientific Directorate, Istituto Nazionale Tumori, IRCCS Fondazione G. Pascale, 80131 Naples, Italy; g.botti@istitutotumori.na.it; 8 Sarcomas and Rare Tumours Unit, Istituto Nazionale Tumori, IRCCS Fondazione G. Pascale, 80131 Naples, Italy

**Keywords:** neuroendocrine tumors, octreotide, Tregs, MDSCs, immune-response modulation

## Abstract

Octreotide long-acting repeatable (LAR) is largely used to treat functional and/or metastatic neuroendocrine neoplasms (NENs). Its effect in controlling carcinoid syndrome and partially reduce tumour burden is attributable to the ability of octreotide to bind somatostatin receptors (SSTRs) on the tumour and metastasis, regulating growth hormone secretion and cell growth. Notably, SSTRs are also expressed, at different levels, on Tregs. Tregs, together with myeloid-derived suppressor cells (MDSCs), are key components in the anti-tumour immunoregulation. This is the first prospective study aimed to explore the impact of Octreotide (OCT) LAR on the immune system, with a particular focus on Tregs and MDSC cells. Here, we show that circulating Tregs are elevated in NENs patients compared to healthy donors and that treatment with OCT LAR significantly decrease the level of total Tregs and of the three functional Tregs populations: nTregs, eTregs and non-Tregs. Furthermore, OCT LAR treatment induces a functional impairment of the remaining circulating Tregs, significantly decreasing the expression of PD1, CTLA4 and ENTPD1. A trend in circulating MDSC cells is reported in patients treated with OCT LAR. The results reported here suggest that the effect of OCT LAR on Tregs could tip the balance of the patients’ immune-system towards a durable anti-tumour immunosurveillance with consequent long-term control of the NENs disease.

## 1. Introduction

Neuroendocrine neoplasms (NENs) are a heterogeneous group of malignancies derived from the neuroendocrine cell compartment [1]. NENs incidence and prevalence are steadily increasing due to the improvement of diagnostic and therapeutic tools; however, the large part of patients presents with advanced and symptomatic disease [2]. While chemotherapy (platinum-based) is the mainstay of treatment for neuroendocrine carcinomas (NECs), somatostatin analogs (SSAs: octreotide and lanreotide) represent the backbone of the treatment for well-differentiated neuroendocrine tumours (NETs). SSAs play a key role in controlling hormone-related symptoms (carcinoid syndrome) and improving clinical benefit [3,4,5]. SSAs mainly act inhibiting tumour growth; this results in a good disease control but in a weak tumour shrinkage effect. In fact, octreotide (OCT) long-acting release (LAR) showed an objective response rate (ORR) of 3–10%, but the disease stabilization rates were up to 50–60% [6]. In addition, OCT LAR (30 mg intramuscular (im) every 28 days) significantly ameliorates the time to tumour progression (TTP) when compared to placebo in either functioning (hormone secreting) or non-functioning (non-secreting) metastatic well-differentiated midgut NETs [4,5], and registered in the PROMID trial a stabilization rate of 60%.

The rationale for the diagnostic and therapeutic use of SSAs in NETs is based on the expression of somatostatin receptors (SSTRs), particularly SSTR2 subtype, in tumour tissue. However, the development of new SSAs and the emerging novel findings on SSTRs physiology has prompted new investigations aimed at developing innovative diagnostic tools and, hopefully, therapeutic approaches. Interestingly, SSTRs are expressed both in peripheral and tumour-infiltrating lymphocytes [7,8], and their expression dynamically depends on the cell trafficking through and within lymphoid structures [9,10,11,12]. In this context, the interactions between SSAs and immune system and the role played by SSAs in the match between neuroendocrine tumours and immunological response are largely unexplored.

The immune response is orchestrated through a complex interrelation between soluble mediators (cytokines, chemokines, etc.) and effector/regulatory lymphocytes. The result is a dynamic balance between activation and the inhibition of the immune response. Many modern drugs aimed to revitalize the anti-tumour immunity [13] act on this balance. Notably, Tregs (regulatory T cells), MDSCs (myeloid-derived suppressor cells) and TAMs (tumour-associated macrophages) are regulatory cells participating in the regulation of immune responses, and their functional excess may contribute to disease progression in some cancers [14,15,16,17,18,19]. Both MDSCs and Tregs expand systematically in the peripheral blood (PB) of preclinical tumour models, and promote T-cell dysfunction that in turn favours tumour progression [19].

Tregs were originally identified as CD4+CD25+ T cells, with crucial roles in maintaining self- tolerance and thus preventing autoimmune disease. Three phenotypically and functionally distinct subsets can be defined based on FOXP3 expression: CD45RA+ FOXP3low naïve or resting Treg (nTreg), CD45RA- FOXP3 high effector or activated Treg (eTreg) and immunosuppressive CD4+ CD45RA- FOXP3low cells [20,21,22,23,24,25]. Furthermore, Tregs express the immunocheckpoint receptors PD-1, CTLA4, CXCR4, GITR and ENTPD1. Regulated compartmentation of PD-1 has been observed to discriminate resting CD4 + CD25 + Treg from activated T cells [25,26,27].

MDSCs are immature, immunosuppressive, myeloid cells that increase in inflammatory diseases, particularly tumours, and suppress anti-tumour immunity [16,28]. MDSCs produce inhibitory factors (e.g., IL-10, arginase) that inhibit T cells and promote Tregs and detrimental M2 macrophages [17]. MDSC can be divided in (i) PMN (polymorphonucleated)-MDSC (CD11b+CD14-CD15+) and (ii) M (monocytic)-MDSC (CD14+HLA-DR-/lowCD15-CD11b+), characterized by distinct suppressive pathways of T cells functions [29]. MDSC immature progenitors, not yet identified in humans, share the majority features with MDSC subsets, and are defined as early-stage MDSC (eMDSC) [29]. PMN-MDSCs are the predominant subset in human tumors [18].

Longitudinal evaluation of peripheral Tregs and MDSC cells has been reported as a surrogate biomarker representing tumour microenvironment (TME). Both MDSCs and Tregs expand systematically in the peripheral blood (PB) of preclinical tumour models and promote T-cell dysfunction that in turn favours tumour progression [19].

MDSCs and Tregs basal values were significantly higher in locally advanced rectal cancer (LARC) patients treated with neoadjuvant chemo-radiotherapy, as compared to healthy donors (HD). Moreover, LARC poor responder patients displayed a significant increase of Treg-PD-1 [30]. With the intent to shed further insights into OCT LAR mechanism of action in NEN patients, the IMMUNeOCT study was designed.

The IMMUNeOCT study is the first prospective, longitudinal study aiming to explore the impact of OCT LAR on Tregs and MDSC population, as detected in the peripheral blood of OCT LAR-treated G1/G2 NENs patients.

## 2. Materials and Methods

### 2.1. Study Design

**Figure 1:**
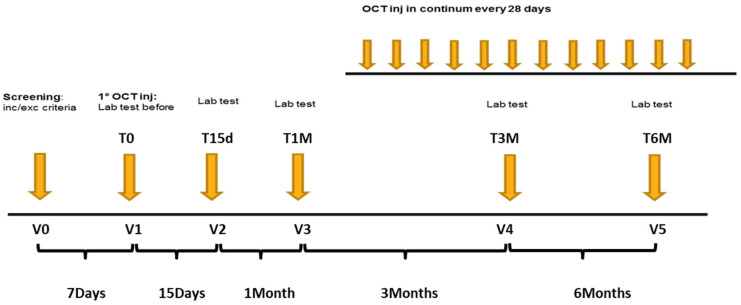
Flow Chart of the study design.

This is a monocentric, interventional, prospective, single-arm study. Peripheral immune phenotypes were analysed in fresh venous blood from healthy donor and from patients over treatment. Patients samples have been collected at the first visit (V1), prior OCT LAR treatment initiation, and at the second (V2), third (V3), fourth (V4) and fifth (V5) visit, at 15 days,1 month, 3 months and 6 months post OCT LAR treatment initiation, respectively. The study design is shown in Figure 1.

The study design was approved by the Ethic Committee of Istituto Nazionale Tumori, IRCSS “Fondazione G. Pascale” of Naples, under protocol number 720/2017.The trial has been registered in the European Union Drug Regulating Authorities Clinical Trials (EudraCT) Database, under the number EudraCT2017-001613-83, and in the clinicaltrial.gov registry under the number NCT04129255.

### 2.2. Patients Population

Patients aged ≥ 18 years with histologically confirmed metastatic GEP-NET and functioning lung-NET and candidate to receive treatment with SSAs were eligible. Between July 2017 and December 2018, 35 patients (pts) were enrolled in the IMMUNeOCT trial at the European Neuroendocrine Tumor Society (ENETs) Centre of Excellence of Naples. Patients characteristics are described in Table 1. All subjects enrolled in the study signed an informed consent form (ICF), were naïve to SSAs treatment, and received OCT LAR intramuscular (im) injection every 28 days the as only medication for NETs treatment. The drug was available as a single-use kit containing 30 mg of octreotide acetate for im injection, and it was stored and protected from light at refrigerated temperatures between 2 °C and 8 °C, until the time of use.

Gallium68 (Ga 68) Dotatoc was performed in all the patients and resulted in being positive for all of them.

Drug administration and all the clinical activities were performed in accordance to good clinical practice (GCP) compliance, with global and local regulatory requirements, protocol and internal SOPs. All adverse events were recorded, starting from the signing of ICF until the follow-up phase, according to Common Terminology Criteria for Adverse Event ver. 4.0 (CTCAE).

### 2.3. Healthy Donors

Healthy donors (HDs) were recruited among blood donors of the Immunohematology and Transfusional Medicine Service at Istituto Nazionale Tumori, IRCSS “Fondazione G. Pascale”. HDs signed an informed consent and screened for evidence of blood infection (HIV, HCV, HBV), renal and hepatic failure.

### 2.4. End Points

Primary study endpoint was to evaluate the peripheral modifications in immune-regulatory cells induced by OCT LAR injection administered every 28 days, in monotherapy from baseline until sixth month of treatment in naïve patients. Secondary endpoint was to evaluate progression free survival (PFS), defined as the time elapsed from first dose administration to the disease progression (PD), evaluated according to RECIST criteria (version 1.1). The immune-response evaluation was performed in peripheral blood samples collected at baseline, on day 14, on day 28, on day 84 and on day 168, in association with the laboratory assessment of haematology, biochemistry and chromogranin-A. Tumour response rate was locally assessed by a triphasic computed tomography (CT) scan at week 12, and then every 12 weeks from the first dose administration date.

### 2.5. Flow Cytometry

Flow cytometry was performed on peripheral blood (collected in EDTA K3 vacutainer tubes) for Tregs evaluation, and on PBMC (human peripheral blood mononuclear cells) for MDSC, obtained from the blood separated by density gradient centrifugation Ficoll^®^ Paque protocol (Sigma-Aldrich, Darmstadt, Germany, Europe). Cells were stained with antibodies for 30 min at 4 °C, and washed with BD wash buffer (PBS/0.2% BSA/0.01% NaN3). Intracellular staining for FOXP3 and CTLA-4 was performed, using a commercially available kit (BD Cytofix/Cytoperm; fixation and permeabilization kit; BD Pharmingen) according to the manufacturer’s instructions. For the identification of Treg cells, the following fluorochrome-labelled monoclonal antibodies (BD Bioscience, San Jose, CA, USA) were used: Horizon-V450 anti-FOXP3(clone259D/C7), Pe anti-CD25(clone 2A3), PercP anti-CD152 (CTLA4) (clone BNI3), Pe-Cy7 anti-CD127 (clone HIL-7R-M21), APC anti-CD279 (PD-1) (clone MIH4), PeCy5 anti-CD184 (clone 12G5), APC anti-CD39(clone TU66), Alexa Fluor647 anti-Helios (clone 22F6) and APC-Cy7 anti-CD4(clone RPA-T4).

For MDSC subsets characterization, the following antibodies were used: FITC anti-Lineage (BD), PE anti-CD11b (clone Mac-1), PER-CP anti-CD33 (clone P67.6), PeCy7 anti-HLA-DR (clone G46-6), APC anti-CD15 (clone HI98), and APC-Cy7 anti-CD14(clone MφP9) (BD Bioscience, San Jose, CA, USA). Viability was analysed using LIVE/DEAD cell stain (Invitrogen, Carlsbad, CA, USA). A minimum of 100,000 events for each sample were collected, and data were analysed using FACSDiva™ 8.0 Software (BD Bioscience).

Data were analysed using GraphPad Prism Software (GraphPad Software, Inc., La Jolla, CA, USA).

### 2.6. Statistical Analysis

Wilcoxon matched pairs signed rank test was used to assess statistical significance. The distribution of the variables was tested with Kolmogorov–Smirnov test. Normally, distributed continuous variables were expressed as mean ± standard deviation (SD), whereas continuous data with skewed distributions were expressed as median (interquartile range (IQR)). Results were considered significant at * *p* ≤ 0.05, ** *p* ≤ 0.005 and *** *p* ≤ 0.0005.

## 3. Results

### 3.1. High Tregs Number in PB of NENs Patients

**Figure 2:**
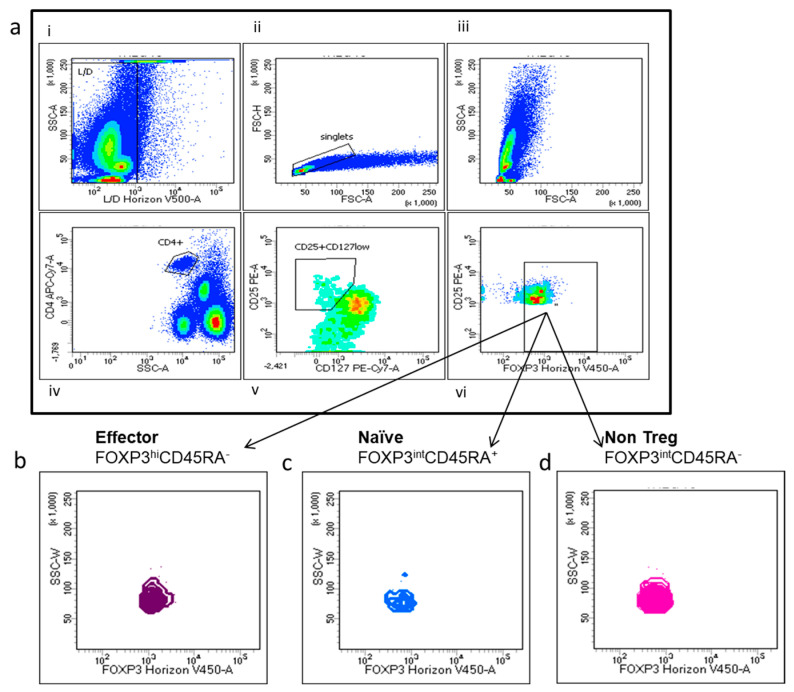
Multi-parametric Tregs and Tregs subtyping gate-strategy. (**a**) (i–vi) Representative fluorescence-activated cell sorting (FACS) plots: (i) dead cells identified as positive Live/Dead Horizon V450 and (ii) doublets cells were excluded; (iii) T lymphocytes were gated on the basis of their scatter parameters and (iv) CD4+ T cells were identified and analysed for CD25 and CD127 surface expression: (v) CD4+ T cells expressing high levels of CD25, negative or low expression of CD127, (vi) and FoxP3 positivity was considered as Treg cells (g–h) Treg subpopulation were identified according to Foxp3 level and CD45RA in eTregs (FoxP3hiCD45RA) (**b**), nTregs(FoxP3intCD45RA+) cells (**c**) and non-Treg intCD45RA- cells (**d**).

**Figure 3:**
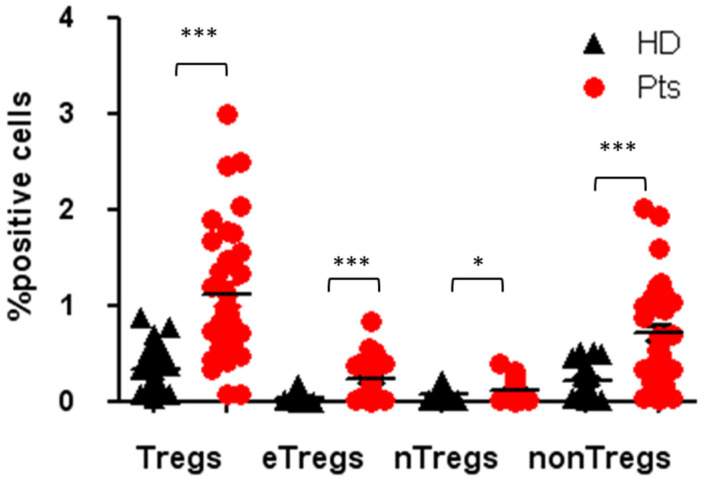
Tregs and Tregs subpopulation in patients and HDs at the baseline. Percentage of circulating Tregs, eTregs, nTregs, non-Tregs in patients and healthy donors, expressed as percentage of cells positives for the relative markers reported in Figure 2. * *p* < 0.05; *** *p* < 0.0005. Pts, patients; HDs, Healthy donors.

Tregs are defined as CD4^+^, CD25^hi^, CD127^low^ and FOXP3^+^. Tregs’ multi-parametric gate-strategy is shown in Figure 2. Tregs comprise dynamic subpopulations: effector Tregs (eTregs) identified as FoxP3^HI^ CD45RA^−^, naïve Tregs (nTregs) as Fox-P3^int^CD45RA^+^ and non-Tregs Fox-P3^int^CD45RA^−^. While the effectors and naïve Tregs displayed specific suppressive function, the non-Treg mainly secreted IL-2, IL17 and γ IFN, displaying a more inflammatory phenotype. Peripheral Tregs were evaluated at T0 in 35 NEN patients and 23 healthy donors (HD). As shown in Figure 3, NEN patients displayed higher number of Tregs at T0, as compared with healthy controls (1.12 ± 0.14 vs. 0.34 ± 0.05) (*p* < 0.0005). All the subpopulation were equally affected in patients as compared to HD eTreg (0.23 ± 0.04 vs. 0.04 ± 0.08) (*p* < 0.0005), nTreg (0.12 ± 0.02 vs. 0.07 ± 0.01) (*p* < 0.05) and non-Tregs (0.71 ± 0.10 vs. 0.22 ± 0.03) (*p* < 0.0005). 

### 3.2. Tregs Decreased during Octreotide LAR Treatment

**Figure 4:**
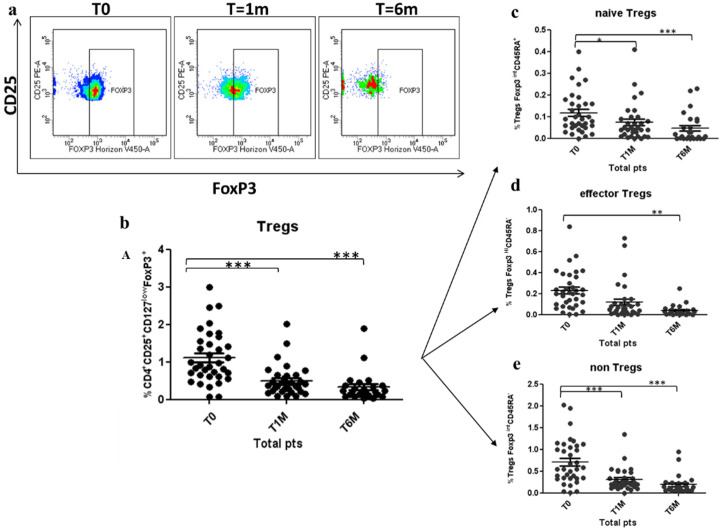
Reduction of Treg cells and Tregs subpopulation during Octreotide LAR treatment: (**a**) Representative sample analysed by flow cytometry, showing the reduction of circulating Tregs in a patient after 1 and 6 months of treatment. (**b**–**e**) Decrease of peripheral Tregs, naïve Tregs, effector Tregs and non-Tregs after 1 month (T1M) and 6 months (T6M) of treatment with Octreotide Lar. Tregs are expressed as percentage of T cells CD4+CD25+CD127lowFoxP3+ (**b**); naïve Tregs are expressed as percentage of Tregs CD127lowFoxP3intCD45RA+ (**c**); effector Tregs are expressed as percentage of CD127lowFoxP3hiCD45RA- Tregs (**d**); and non-Tregs are expressed as percentage of CD4+CD25+CD127lowFoxP3intCD45RA-. * *p* < 0.05; ** *p* < 0.005; *** *p* < 0.0005.

A longitudinal Tregs evaluation was conducted in 35 patients during the octreotide LAR treatment. Tregs were significantly decreased by 55% after 1 month of treatment (1.12 ± 0.14 vs. 0.50 ± 0.08; *p* < 0.0005), and by 71% after 6 months of treatment (1.12 ± 0.14 vs. 0.32 ± 0.08; *p* < 0.0005) (shown in Figure 4a,b). There was not statistical difference between groups, as shown in Table 1. The three functional Tregs subpopulations, naïve Tregs, active Tregs and non-Tregs, were concomitantly and significantly reduced by 58% (0.12 ± 0.02 vs. 0.05 ± 0.01; *p* < 0.0005), 61% (0.23 ± 0.04 vs. 0.09 V 0.05; *p* < 0.005) and 73% (0.71 ± 0.10 vs. 0.19 ± 0.04; *p* < 0.0005), respectively, after 6 months of octreotide LAR treatment (shown in Figure 4c–e). The results of Tregs level at 3 months were consistent with the gradual dynamic reduction shown over time.

With the intent to characterize Tregs’ functional status, the expression of immuno-checkpoint and immuno-markers PD-1, CTLA4, CXCR4, ICOS, Helios and ENTPD1 was evaluated on Tregs/Tregs subpopulations. 

**Figure 5:**
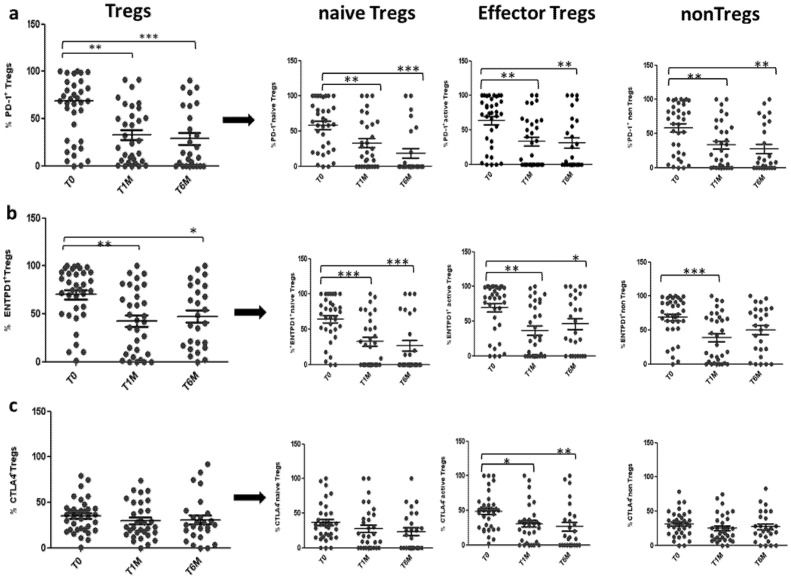
PD-1, ENTPD1, CTLA4 expression on Tregs during OCT LAR treatment: Expression of PD-1 (**a**), ENTPD1 (**b**) and CTLA4 (**c**) in Tregs and Tregs subpopulation, before (T0), after 1 month (T1M) and after 6 months (T6M) of OCT LAR treatment. * *p* < 0.05; ** *p* < 0.005; *** *p* < 0.0005.

There was a significant decrease in the percentage of total Tregs, expressing PD-1 and ENTPD1 at 1 month and 6 months of treatment with OCT LAR. The total Tregs expressing PD-1 and ENTPD1 decreased by 44% (59.4 ± 6.6% vs. 33 ± 6%; *p* < 0.005) and 39% (69.9 ± 5.7% vs. 42.4 ± 6,7%; *p* < 0.005) after 1 month of treatment, and by 52% (59.4 ± 6.6% vs. 28.7 ± 0.06%; *p* < 0.0005) and 33% (69.9 ± 5.7% vs. 47.1 ± 0.07%; *p* < 0.05), after 6 months of OCT LAR treatment (shown in Figure 5a,b). This decrease was paralleled by a significant decrease in PD1 and ENTPD1 expression in the three functional subpopulation, nTregs, eTregs and non-Tregs, after both 1 month and 6 months of treatment (shown in Figure 5a,b). Conversely, the expression of CTLA4 was specifically and significantly decreased only in the eTregs population, the most suppressive population, with a reduction of 37% (48.6 ± 5.3% vs. 30.7 ± 5.5%; *p* < 0.05) and 45% (48.6 ± 5.3% vs. 26.5 ± 0.06%; *p* < 0.005) after 1 month and 6 months of treatment, respectively (shown in Figure 5c).

CXCR4, ICOS and Helios expression were not significantly modified over treatment (data not shown).

OCT LAR treatment significantly reduces Tregs number and function in NEN patients, suggesting a direct effect of OCT LAR on immune regulatory cells.

### 3.3. Peripheral MDSCs Subsets Evaluation

The MDSCs consist of immature myeloid cells and have a bewildering diversity of phenotypes.

**Figure 6:**
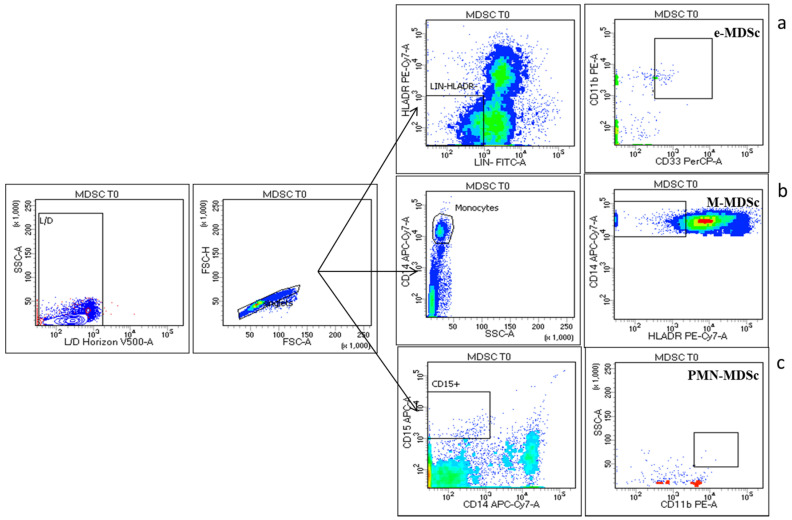
MDSC Cytometry gating strategy: three major MDSC subsets have been identified: The early MDSC (e-MDSC) was identified with surface expression of LIN-HLADR-/lowCD11b+CD33+ (**a**) myeloid MDSC (M-MDSC) as CD14+HLADRlow (**b**) and polymorphonuclear MDSC (PMN-MDSC) as CD15+CD11b+ SSCAhigh (**c**) gated in Live (Live/Dead Horizon V450 negative cells) and singlet cells.

**Figure 7:**
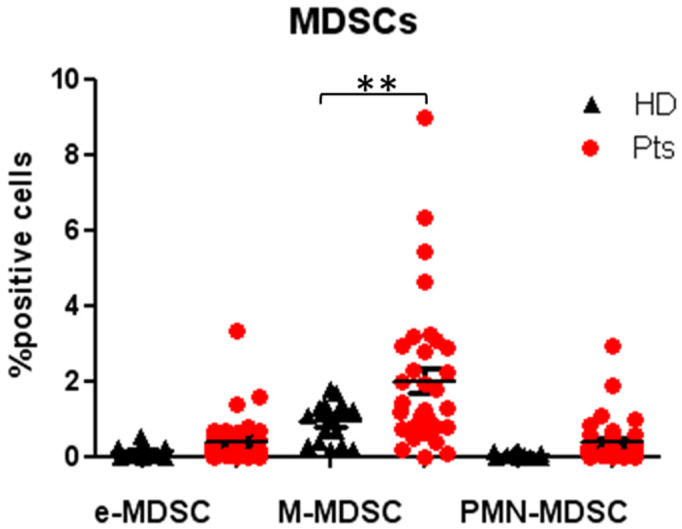
Evaluation of MDSCs subsets in patients and HDs at baseline: Percentage of circulating early MDSC (e-MDSC), myeloid MDSC (M-MDSC) and polymorphonuclear MDSC (PMN-MDSC) in patients and healthy donors, expressed as percentage of cell positives for the relative markers reported in Figure 6. ** *p* < 0.005. Pts, patients; HDs, Healthy donors; MDSC, myeloid-derived suppressor cells.

The strategy gates used to evaluate the MDSC subsets are the standardized strategy used by Bronte et al. [28], that had defined three important subsets of MDSCs, myeloid MDSC (M-MDSC), with expression of CD14^+^HLADR^low^, early MDSC (e-MDSC) as LIN^-^HLADR^-/low^CD11b^+^CD33^+^ and MDSC (PMN-MDSC) as CD15^+^CD11b^+^ SSCA^high^ (shown in Figure 6). The median frequencies of the three MDSC populations were higher in NENs patients, as compared to HDs M-MDSC (1.99 ± 0.38 vs. 0.27 ± 0.02) (*p* < 0.005), e-MDSC (0.41 ± 0.13 vs. 0.01 ± 0.001), PMN-MDSC (0.37 ± 0.12 vs. 0.02 ± 0) (shown in Figure 7). The longitudinal study in patients revealed a trend in the decrease of peripheral blood MDSCs.

### 3.4. Clinico-Pathological Associations with Tregs and MDSCs Dynamics

With the intent to correlate the peripheral Tregs basal value and their OCT LAR induced decrease with clinical pathological patients features, known clinical prognostic factors were analysed. No significant correlation was detected between the peripheral Tregs basal value and their OCT LAR induced decrease and metastasis site (hepatic vs. other sites) and number, Ki67 (Ki67 < 5 vs. Ki67 > 5), grading of tumour (G1 vs. G2), size of the tumour and functional status (functional vs. non-functional), suggesting that, although Tregs decrease may represent the OCT LAR benefit, this is not enough to impact on known prognostic factors.

There were no progressions during the observation period of 6 months. Two patients experienced a partial response (PR), whereas most of the patients, 33 patients, had a stable disease (SD) after 6 months of treatment with SSAs. The overall response rate (ORR) was 6%.

## 4. Discussion

In this manuscript, the impact of OCT_LAR treatment was evaluated on Tregs and MDSC cells on NEN patients. Peripheral Tregs cells were significantly higher in NEN patients as compared to healthy donors, and the OCT LAR treatment decreased Tregs in term of percent and function.

The MDSCs and Tregs orchestrate a complex immunosuppressive network that works to ensure self-tolerance, but that also suppresses the antitumor immunity, ultimately causing cancer development and progression. 

An increased level of circulating MDSCs and Tregs has been associated with advanced stage and poor prognosis in different cancers [31,32,33,34]. However, little is known on the expression and potential role of circulating MDSCs and Tregs in NENs. Vikman et al. demonstrated that the number of circulating Tregs was significantly higher in patients with midgut carcinoids compared to healthy population, and that the levels of circulating FOXP3+ cells were proportional to the tumour burden [35]. Here, we showed a significant 3.3-fold increase of Treg cells’ level in patients with GEP-NET and Lung NET in comparison to HDs. A significant increase was revealed for the three subpopulations of Tregs analysed; however, the highly suppressive eTregs subpopulation mostly increased, as compared to nTregs and non-Tregs.

A trend of increase was shown in circulating MDSCs cells in patients compared to HDs, but it was significant only for the M-MDSCs subpopulation. In our study, we did not notice a correlation between the level of Tregs at the baseline and tumour burden and/or tumour site, this may be due to the fact that in this study were included patients with mid gut tumours, but also with NETs in other GI sites and Lung NETs.

Several reports have described a correlation between a decrease in circulating Tregs numbers and better outcome during anti-cancer treatment [36,37,38]. However, there was no experience on the effect of SSAs on the immune system. In this study, we showed a significant reduction in Tregs and Tregs subpopulation, eTregs, nTregs and non-Tregs, in metastatic GEP-NET and lung-NETpatients during treatment with OCT LAR. As expected, PFS and ORR are not significantly correlated to Tregs decrease. However, it should be considered that the treatment with SSAs in Lung and GEP-NET is frequently associated to long PFS and low response rates [4,5,6]; for this reason, the timepoint of six months might have been too early to evaluate any time-to-outcome. No toxicity and no severe adverse events are recorded during the six months of treatment.

A decrease in the Treg PD-1 and ENTPD1 expression was also reported, showing that OCT LAR not only reduces the level of the peripheral Tregs, but also induces a functional impairment of the Tregs subpopulations.

PD-1-PD-L1 axis is crucial to promote nTregs conversion into eTregs, to regulate the eTregs stability in peripheral tissue, and to enhance and maintain the immunosuppressive function of eTregs on effector T cells (Teff) [39]. Thus, the PD-1 depletion on circulating Tregs will suppress eTregs expansion and function, ultimately releasing the immunosuppressive brake on Teff cells.

ENTPD1 (CD39) is the main ectonucleotidase expressed in human Tregs. However, ENTPD1 expression on Tregs is variable among healthy people, whereas it seems to be increased in patients with cancer [40].

Via the ENTPD1/CD39-CD73-adenosine pathway, Tregs inhibit Teff proliferation, NK cells cytotoxic activity and cytokine production [41,42]. In addition, the adenosine produced through the ENTPD1-CD73-adenosine pathway drives monocytes differentiation towards an aberrant differentiation in DCs secreting pro-tumorigenic factors [43]. Lastly, CD39 mediates ATP depletion, ATP is an important inhibitor of tumour cells proliferation and a key chemo-attractant of antigen-presenting cells at the tumour site; consequently, its depression leads to an increased proliferation in tumour cells and a decreased anti-tumour immune response [40,44]. Altogether, these results suggest that ENTPD1/CD39 on Tregs has a key immunosuppressive role that ultimately promote tumour growth. Tregs CD39 null lost immunosuppressive function and failed to inhibit Teff cells [40,45]. Therefore, the OCT LAR induced reduction of Tregs ENTPD1 positive, which we reported in our study, results in less functional Tregs, and may restore the Teff and NK cells anti-tumour response, as well as allow the accumulation of ATP at the tumour site that will trigger the recruitment of DCs, monocytes and macrophages at the tumour site.

In this study, we also showed that six months of OCT LAR treatment significantly reduces the percentage of eTregs expressing CTLA-4. CTLA-4 is expressed by eTregs constitutively, and it is specifically essential for eTregs immunosuppressive function [21]. Consequently, we can hypothesize that the OCT LAR-induced loss of CTLA-4 eventually results in a functional breakdown of the eTregs.

The somatostatin receptors SSTR (2, 3, 1 and 4) are expressed on the T lymphocytes [8], and seem to have an immunoregulatory function [7,8,46]. Although there are still some contradictory results in literature on the role of SSAs in the modulation of the immune system, the majority of the results seem to point out that somatostatin and/or somatostatin analogs enhance T cells cytotoxic activation, through the adhesion of T cells to fibronectin, collagen type IV, laminin, and β_1_ integrins [12,47]. In addition, somatostatin and SSAs promote T cells differentiation into lymphokine-activated killer cells, enhance the cytolytic lymphokine-activated killer activity induced by IL-2, through the inhibition of adenylate cyclase and activation of protein kinase C [48], and induce the secretion of IL-2, IL-4, IL-10, and interferon-γ in T helper cells [49].

We can therefore postulate that the effects on circulating Tregs, observed in this study, are due to a direct effect of OCT LAR on Tcells SSTRs. However, SSAs’ direct effect on Tregs may act along with an effect on NETs cells, and on their relative profile of cytokine and chemokine secretion.

**Figure 8:**
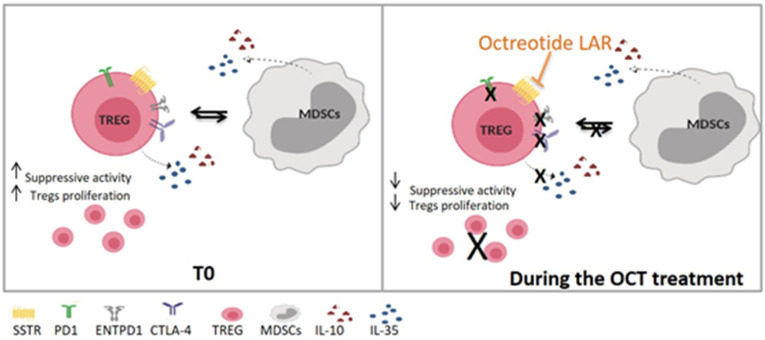
Proposed OCT LAR mechanism of action on immune cells. T0, Time 0—before Octreotide treatment; OCT, Octreotide.

A trend in MDSC decrease during OCT LAR treatment was also observed in this study. We can speculate that the MDSC reduction is secondary to the Tregs inhibition that causes a reduction in cytokines production (e.g., IL10 and IL35), consequently inhibiting the accumulation and function of MDSC (shown in Figure 8). We are currently testing this hypothesis, evaluating cytokines’ levels in SSAs treated patients’ blood and the functional phenotype of the circulating MDSC in the same group of patients.

Immunologic therapy is the new frontier of anti-cancer treatment, and immune checkpoint inhibitors have rapidly enriched the therapeutic scenario of many cancers (melanoma, NSCLC, lymphomas, renal cancer, etc.) PD-1/PD-L1 and CTLA4/B7 axes are crucial under normal conditions to protect normal cells from T cells recognition (self-tolerance and exhaustion). In fact, the engagement of PD-L1 and/or B7 (expressed by normal cells) delivers a potent inhibitory signal in T cells. These signals contribute, along with the cellular regulatory counterpart (Tregs and MDSCs), to the “fine tuning” of the immune response. However, numerous types of cancers upregulate PD-L1 and/or B7 proteins to “escape” T-cell mediated recognition and elimination. The observations that NETs express both PD-L1 and B7 protein and that they are infiltrated by CD3+ T cells [50,51] prompted the design of phase II/III clinical studies aiming to assess the efficacy of immune checkpoint inhibitors (durvalumab, pembrolizumab, PDR001, tremelimumab, etc.) in this clinical setting. A complete dissertation of the trials is beyond the scope of this work. However, definitive results are still not published. Considering our data, the assessment of Tregs and MDSCs, particularly in patients undergoing to SSAs, could add value to the predictive power of such trials.

The limitation of our study is that Tregs in the tumour microenvironment have not been evaluated, so we can only speculate that the changes obtained in circulating Tregs parallel the changes in tumour-associated Tregs.

Confirmation of this parallelism in future studies, would be a key finding leading to the recognition of OCT LAR as a positive modulator in the anti-tumour response. This will support OCT LAR as an ideal partner for combined therapy in NENs, as well as provide the rationale of using OCT LAR as long-term maintenance therapy, even after progression.

## 5. Conclusions

The here reported double effect of OCT LAR of reducing circulating Tregs level and impairing their functional profile, together with the well-known anti-tumour growth effect, could be responsible for tipping the balance between the host immune system and the NENs disease towards a long-lasting anti-tumour immunosurveillance, ultimately resulting in a long-term control of the NENs disease. This new balance promoted by OCT LAR might augment the efficacy of a concomitant immunotherapy or targeted therapy in NENs treatment, suggesting a new potential role of OCT LAR, also beyond disease progression, in a combined therapies scheme.

## Figures and Tables

**Table 1:** Comparisons of patient characteristics and Tregs reduction over 6 months of somatostatin analogs (SSAs) treatment.

Characteristics	*n* (%)	Tregs Reduction from T0 to T6M, Median [IQR]	*p*
**Age (Years)**			
Median	59		
Range	33–81		
**Sex**			
Male	18 (51)	0.78 [0.48–1.23]	0.45
Female	17 (49)	0.59 [0.29–0.78]	
**Primary tumour site**			
Lung	7 (20)	0.58 [0.48–1.09]	0.84
GEP	28 (80)	0.62 [0.41–1.22]	
Stomach	11 (31)		
Pancreas	8 (23)		
Ileus	5 (14)		
Rectum	3 (9)		
Mesenteric Nodes	1 (3)		
**Functional Status**			
Functioning	13 (37)	0.65 [0.49–1.30]	0.61
Non-Functioning	22 (63)	0.58 [0.40–1.17]	
**Metastatic Sites**			
Liver	23 (66)	0.65 [0.4–1.03]	0.91
Others	12 (34)	0.56 [0.31–1.38]	
**Metastatic sites n.**			
1	25 (71)	1.08 [0.28–1.5]	0.91
>1	10 (29)	0.6 [0.42–0.85]	
**NET Grade**			
G1	25 (71)	0.66 [0.42–1.23]	0.64
G2	10 (29)	0.58 [0.29–0.78]	
